# Molecular Biodiversity in *Fusarium subglutinans* and *F. temperatum*: A Valuable Tool to Distinguish the Two Sister Species and Determine the Beauvericin Chemotype

**DOI:** 10.3390/jof10110785

**Published:** 2024-11-13

**Authors:** Antonia Susca, Alessandra Villani, Miriam Haidukowski, Filomena Epifani, Antonio F. Logrieco, Antonio Moretti

**Affiliations:** 1 Institute of Sciences of Food Production, National Council of Research (ISPA-CNR), 70126 Bari, Italy; miriam.haidukowski@ispa.cnr.it (M.H.); filomena.epifani@ispa.cnr.it (F.E.); antonio.logrieco@ispa.cnr.it (A.F.L.); antonio.moretti@ispa.cnr.it (A.M.); 2 Institute of Biomanufactoring, Xianghu Laboratory, Hangzhou 310025, China

**Keywords:** SNPs, mycotoxins, biosynthetic gene cluster, maize

## Abstract

*Fusarium subglutinans* and *F. temperatum* are widely distributed maize pathogens recognized as distinct species with a species-specific chemotype based on patterns of mycotoxins. Recent comparative genomic analysis revealed that genomes of both species carry a complete beauvericin (*Bea*) biosynthetic genes cluster, but the key gene *Bea1* in *F. subglutinans* is not functional likely due to a large insertion (NRPS22ins) and multiple mutations (SNP298 and SNP528). We used the recently published genome sequences for these species to develop PCR markers for investigating the distribution of three main mutations in the *Bea1* gene in a large collection of strains of both species from around the world. We also designed a PCR assay for a rapid and reliable discrimination of both species in the evaluation of crop exposure to mycotoxins. Overall, our results showed that SNP528 was the most common mutation, followed by NRPS22ins and SNP298. Moreover, phylogenetic analyses suggest that non-synonymous SNPs have occurred first, and that the resulting inactivation of BEA production has caused the accumulation of other polymorphisms, including the NRPS22ins, in the entire gene-coding region. The screening for genetic differences between these species could guide future crop management strategies.

## 1. Introduction

Species within the genus *Fusarium* are of agricultural, medical, and food/feed safety concern, due to their toxigenicity and worldwide distribution [1,2]. The accurate species-level identification as well as knowledge of the host range, geographic distribution, and mycotoxin production potential of each fungal species is of paramount significance to correctly evaluate the mycotoxicological risks and set up prediction models for crop management, even more so in the current scenario of the imminent challenges posed by climate change and food security. Indeed, the importance of surveys on *Fusarium* biodiversity is also related to the changes in environmental conditions worldwide, which significantly influence their distribution on food crops and the related toxicological profile of species [1,3,4,5].

The most recent evolution of species concepts obtained through multilocus molecular systematic studies and advances in Next-Generation Sequencing technologies are providing tremendous insights into the biodiversity of *Fusarium*, the taxonomic relationships within the genus and the genetic potential of each species to produce diverse secondary metabolites (SMs). A deeper understanding of biosynthetic gene clusters and species concepts has highlighted several confounding issues, which has fostered a collective effort toward the filling of missing identification details and the correct reappraisal of mycotoxigenic potential within *Fusarium* [2,6,7,8,9,10].

*Fusarium subglutinans* and *F. temperatum* are important mycotoxin-producing pathogens of maize [2] belonging to the American clade of the *F. fujikuroi* species complex (FFSC). In the last decade, new data obtained by multilocus phylogeny [11,12] and genomic analyses [12,13] led to the recognition of these two entities, previously identified as cryptic lineages, as different species with likely semipermeable boundaries. Two recent studies focused on SMs’ variation and gene flow between *F. subglutinans* and *F. temperatum*. The first study analyzed the chemotypes and genotypes for strains from both species to analyze the ability to produce beauvericin (BEA), revealing that genomes of both species have the presence of a complete *Bea* biosynthetic gene cluster in all genomes [13]. A deeper analysis at the sequence level highlighted that the *Bea1* gene, encoding the nonribosomal peptide synthase responsible for synthesizing the beauvericin backbone (NRPS22), was not functional in *F. subglutinans* due to a large insertion and multiple mutations. The evolutionary analysis investigated the divergence between *F. subglutinans* and *F. temperatum*, highlighting a gene flow from *F. subglutinans* into *F. temperatum*, greater than the gene flow in the reverse direction, supporting the evidence of semipermeable boundaries between these two species [14]. Furthermore, the analyses showed that the number of polymorphisms shared between the two species was greater near the telomeres of all 12 chromosomes, where most of the genes involved in mycotoxin and plant–pathogen interactions were located, as also highlighted in other fungal genomes [15,16].

Although there is evidence that aberrant versions of the *Bea1* gene occur in *F. subglutinans*, while an apparently functional copy of the rest of *Bea* gene cluster is present both in *F. subglutinans* and *F. temperatum*, it would be interesting to determine whether those mutations are spread among *F. subglutinans* strains, as well as in those *F. temperatum* strains that do not produce BEA. The current study evaluates the worldwide biodiversity of these two species, focusing on the *Bea1* gene and related DNA variabilities, responsible for its inactivation in *F. subglutinans*, as previously assessed by comparative genomic analyses in a few species-representative strains [13]. Furthermore, we developed a practical tool for species identification and for assessing BEA production risk. Specifically, we carried out three key activities: (i) the development of species-specific PCR primers based on the elongation factor 1α gene (*Tef1*), for rapid and reliable discrimination of the two morphologically undistinguishable species; (ii) development of PCR primers targeting the *Bea1* gene, which are useful to screen for the three DNA variations responsible for gene inactivation in a large set of *F. subglutinans* and *F. temperatum* strains; and (iii) DNA sequencing and phylogenetic analyses of screened traits of the *Bea1* gene to hypothesize the evolutionary history of these mutations. This work provides new knowledge about nucleotide biodiversity in *F. subglutinans* and *F. temperatum*, *specifically* in *Bea1* and *Tef1*, and exploits it to develop molecular tools applicable for diagnostic purposes, helpful in agricultural crop and mycotoxin risk management.

## 2. Materials and Methods

### 2.1. Fungal Strains

A set of 92 fungal strains (48 *F. subglutinans* and 44 *F. temperatum*), taxonomically identified in previous studies [10,12,13,17] and isolated from maize samples collected from several geographical origins (Argentina, Austria, Belgium, Germany, the USA, Italy, Netherland, Poland, Slovakia, Switzerland, Yugoslavia, Turkey) were examined in this study (Table S1). Strains were retrieved from the Agri-Food Toxigenic Fungi Culture Collection [18] at the Institute of Science of Food production (ISPA; Bari, Italy) and from the private (RC) collection of the Universidad Nacional de Rio Cuarto (Argentina).

### 2.2. DNA Extraction

A suspension of spores, from each fungal strain, was grown in Wickerham medium, containing 40 g glucose, 5 g peptone, 3 g yeast extract, 3 g malt extract, and water up to 1 L, for mycelium production. Mycelia were filtered, lyophilized, and subjected to mechanical grinding using 5 mm iron beads in a Mixer Mill MM 301 (Retsch GmbH, Haan, Germany) for DNA extraction. Total genomic DNA was extracted using the “Wizard^®^ Magnetic DNA Purification System for Food” kit (Promega, Madison, WI, USA) according to the manufacturer’s protocol, using 10 mg of lyophilized mycelium. The quality of genomic DNA was determined by 0.8% agarose gel electrophoresis and quantification with spectrophotometric analysis using NanoDrop ND-1000 (Thermo Fisher Scientific, Waltham, MA, USA). 

### 2.3. Species-Specific PCR Primer Design

Two primer pairs, subF/subR and tempF/tempR, specific for *F. subglutinans* and *F. temperatum*, respectively (Table 1), were designed based on the housekeeping gene translation elongation factor-1 alpha (*Tef1*), using sequences generated in a previous study as a reference [Fumero et al., 2020] [13].

Species-specific primers were designed using Primer Express 3.0 software (Applied Biosystem, Forster City, CA, USA). The PCR reactions containing species-specific primers were performed in 15 μL reactions set up with 200 µM dNTP, 300 nM for each primer, 0.375 U of HotMaster Taq DNA Polymerase (5 Prime), and 20 ng of fungal DNA. Amplifications were performed using the following PCR conditions: denaturation at 95 °C for 5 min; 25 cycles of denaturation at 95 °C for 50 s; annealing at 59 °C for 20 s; extension at 72 °C for 1 min; and final extension at 72 °C for 7 min, followed by cooling at 4 °C until recovery of the samples. Amplification products were checked on 2% agarose gel and visualized with GelRed™ (Biotium, Fremont, CA, USA) under ultraviolet illumination.

### 2.4. Bea1 Gene PCR Primer Design

The public genome sequences of *F. subglutinans* strains RC 298 (JAAIFR000000000) and RC 528 (JAAIFQ000000000), and *F. temperatum* strains RC 2914 (JAAIFN000000000) and CMWF389 (LJGR00000000), were used for the primer design. Three primer pairs targeting the non-ribosomal peptide synthase NRPS22 (*Bea1*) were designed spanning the three variabilities: the 184 bp additional trait, the transition (C→T) at nucleotide position 7685, and the insertion (C) at nucleotide position 5875, hereafter named Nrps22ins, SNP528, and SNP298, respectively (Table 2).

Oligonucleotide primers were constructed and checked for their physical features such as melting temperature, self-complementarity, and secondary structures using Primer Express 3.0 (Applied Biosystems, Foster City, CA, USA) following the rules for PCR primer design. Amplifications were carried out in 15 μL of standard PCR buffer containing 0.2 mM of each dNTP, 300 nM each primer, 0.25 U of Hot Master Taq DNA Polymerase (Fisher Molecular Biology, Rome, Italy), 1× Hot Master Taq DNA Polymerase buffer with 25 mM Mg^2+^, and approximately 20 ng of DNA. The amplification program consisted of an initial 2 min denaturation at 95 °C followed by 35 cycles of 30 s at 95 °C, 30 s at 58 °C, 50 s at 72 °C, and a final 5 min extension step at 72 °C.

### 2.5. RNA Isolation and Reverse Transcription

One representative *F. subglutinans* strain for each nucleotide variability described in the *Bea1* gene (Nrps22ins, SNP528, and SNP298) as well as four BEA-nonproducing *F. temperatum* strains were used for RNA isolation and transcript analysis. Total RNA was extracted using the RNeasy Plant Mini Kit (Qiagen, Milan, Italy), according to the manufacturer’s protocol, and stored at −80 °C. Around 3 µg RNA was used to synthesize first-strand cDNAs using oligo (dT), random hexamers, and SuperScript™ III First-Strand Synthesis system (Life Technologies, Carlsbad, CA, USA) according to the manufacturer’s instructions.

### 2.6. Sequence Analysis

Species phylogeny was investigated by amplifying and sequencing the housekeeping gene translation elongation factor-1 alpha (*Tef1*), using PCR conditions and primers described by O’Donnell et al., 1998 [19]. After amplification, the PCR products were purified with the enzymatic mixture EXO/FastAp (Exonuclease I, *Escherichia coli*/FastAP thermosensitive alkaline phosphatase, Thermo Fisher Scientific Baltics, Vilnius, Lithuania). Both strands were sequenced with the Big Dye Terminator Cycle Sequencing Ready Reaction Kit, purified by gel filtration through SephadexG-50 (Amersham Pharmacia Biotech, Piscataway, NJ, USA) and analyzed on the 3730xl DNA Analyzer (Applied Biosystems, Foster City, CA, USA). Alignment of the two DNA strands was performed using the software package BioNumerics 5.1 (Applied Maths, Sint-Martens-Latem, Belgium), with manual adjustments where necessary.

### 2.7. Phylogenetic Analysis

Sequences were aligned using MUSCLE as implemented in MEGA version X [20] and the resulting alignments were used to infer maximum likelihood trees using IQ-Tree (version 1.6.7) with 1000 bootstrap replicates [21]. The 92 strains were screened for the presence/absence of mutations in the *Bea1* gene by amplifying and sequencing the three loci with primer pairs designed in this study.

## 3. Results

### 3.1. Species Phylogeny

**Figure 1:**
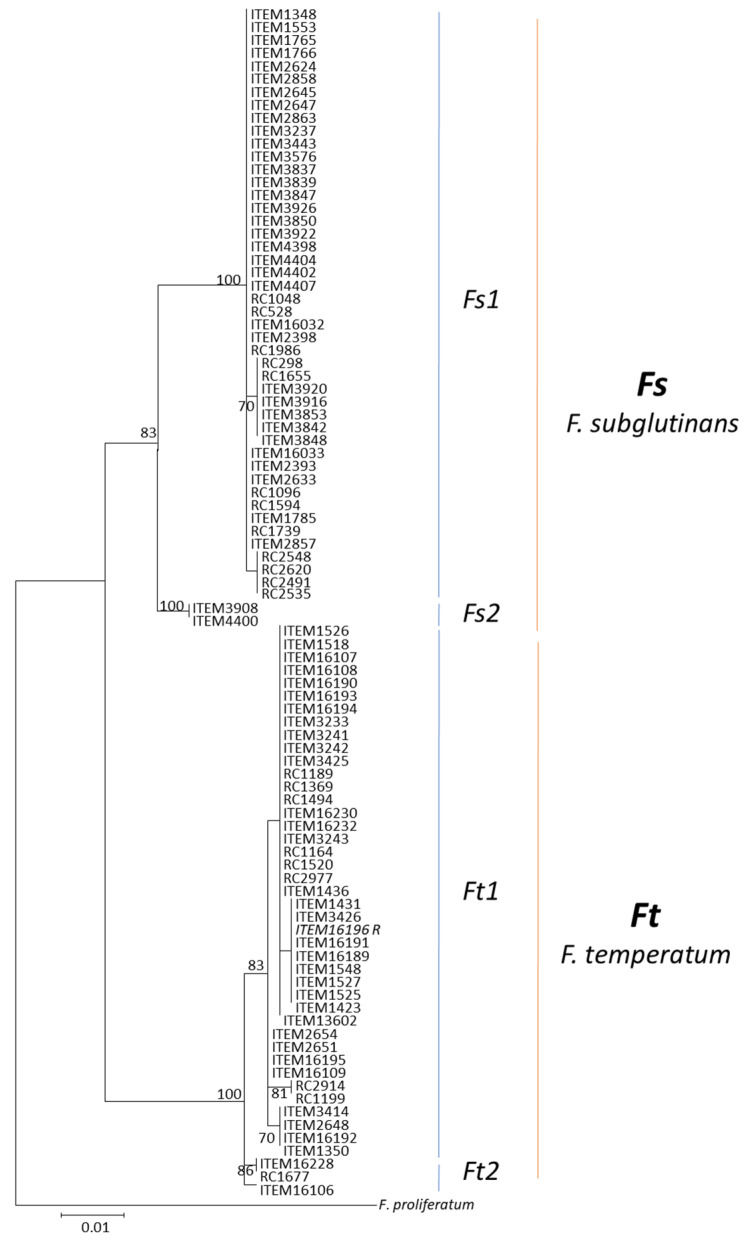
Species tree inferred by ML analysis of *Tef1* gene. Values on branches indicate bootstrap values based on 1000 replicates. Reference isolates are indicated by R. The tree is rooted with *F. proliferatum* (NRRL 62905) according to Fumero et al.’s (2020) study [13].

In the Tef1 phylogeny, the 92 strains were resolved into two distinct and well-supported clades (bootstrap 83 and 100) corresponding to *F. subglutinans* (Fs) and *F. temperatum* (Ft) species, respectively (Figure 1). Subclades within each clade were observed, highlighting further variability within each species.

In the Fs clade, two isolates (ITEM 4400 from Yugoslavia and ITEM 3908 from the USA) nested within the subclade Fs2 (bootstrap 100), while the subclade Fs1 contained all remaining *F. subglutinans* isolates originating from Argentina, Germany, the USA, Italy, Poland, Slovakia, Turkey, and Serbia. In the Ft clade, most of the isolates, including the relative reference strains (40 out of 44) grouped with the subclade Ft1 (bootstrap 83), while subclade Ft2 included three isolates originating from Italy, the USA, and Argentina.

### 3.2. Identification with Species-Specific PCR Primers

**Figure 2:**
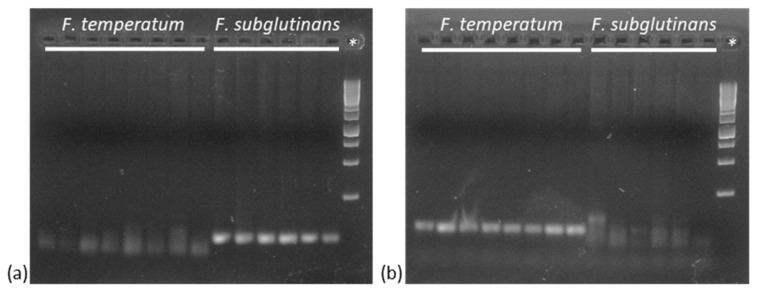
PCR amplifications with species-specific primer pairs targeting (**a**) *F. subglutinans* (subF/subR) and (**b**) *F. temperatum* (tempF/tempR). * GeneRuler 1 kb DNA Ladder.

The optimal PCR conditions and annealing temperature were determined across a range of annealing temperatures (57–60 °C) using a thermal cycler with temperature gradient block. Species specificity was assessed among the two species (Figure 2) and against non-target species, such as *F. proliferatum*, *F. verticillioides*, *F. fujikuroi*, *F. oxysporum*, and *F. globosum*. Both primer pairs generated species-specific amplifications with a PCR fragment of the predicted length. Isolates of non-target fungal species were not detected using the species-specific primer pair.

### 3.3. Bea1 Variability Analysis

The presence of three specific nucleotide variabilities in the Bea1 gene was analyzed by amplifying and sequencing regions including SNP298, SNP528, and NRPS22ins, respectively. The presence or absence of NRPS22ins was first tested by amplifying all 92 strains and comparing the amplicon size on agarose gel. Considering the presence(+)/absence(−) of the pattern NRPS22−SNP528−SNP298, six combinations (I–VI) were observed: (I) + − −, (II) + + −, (III) + − +, (IV) − + −, (V) − − +, and (VI) − − −, while none of the strains showed combinations (VII) − + + and (VIII) + + + (Table 3).

**Figure 3:**
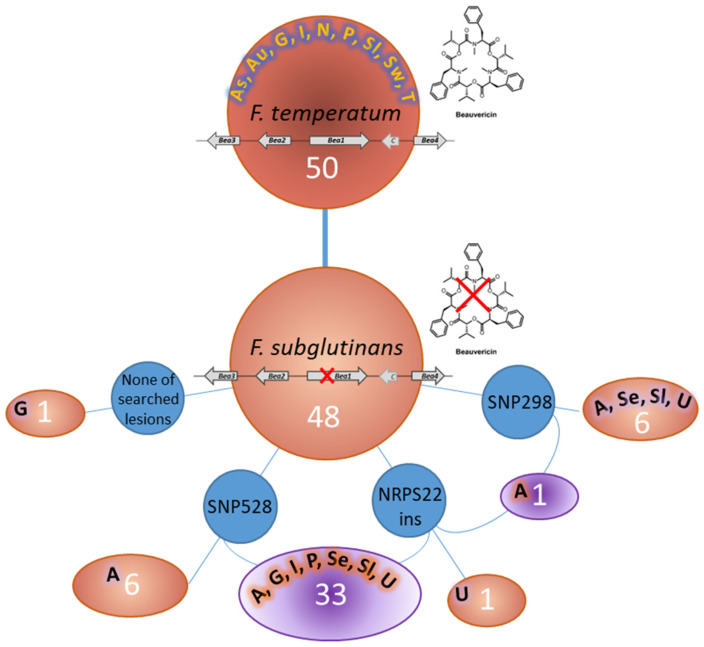
Microevolution in the beauvericin gene cluster of *F. subglutinans* and *F. temperatum*. White numbers in the circles indicate the number of strains. The black letters with colors alone indicate the geographical origin: A: Argentina, As: Australia, Au: Austria, G: Germany, I: Italy, N: Netherland, P: Poland, Se: Serbia, Sl: Slovakia, Sw: Switzerland, T: Turkey, U: USA.

A simplified scheme allows the visualization of different combinations detected in the isolated strains, showing data regarding Bea1 variability and the number of related affected strains, isolated worldwide (Figure 3).

Within the set of *F. subglutinans* strains included in this study, 35 were found to contain the NRPS22ins, 7 the SNP298, and 39 the SNP528. The presence of NRPS22ins (combination I) was observed in one strain collected in Iowa, USA. A combination of NRPS22ins and SNP298 (II) was observed in one strain originated from Argentina, while the presence of NRPS22ins and SNP528 (combination III) was observed in 33 strains derived from Argentina, Germany, Italy, Poland, Serbia, Slovakia, and the USA. SNP298 (combination IV) was observed in six strains collected in Argentina, Serbia, Slovakia, and the USA, while combination V (presence of SNP528) was detected in six strains, all originated from Argentina. The absence of nucleotide variabilities (combination VI) was observed only in one strain collected in Germany (Table 3, Figure 3).

In *F. temperatum*, the Bea1 gene had not the 184 bp insertion (NRPS22ins) both in BEA-producing and -nonproducing strains (ITEM 1518-3233-3243-3426). In *F. subglutinans*, none of the tested strains are BEA-producing, but only some, the strains belonging to combination I (ITEM 3848), II (RC 298), and III (ITEM 3443), present the 184 bp insertion. When present in the gene, the insertion is also kept in transcripts, even though it was expected to act as intron by in silico analysis.

### 3.4. Bea1 Phylogeny

**Figure 4:**
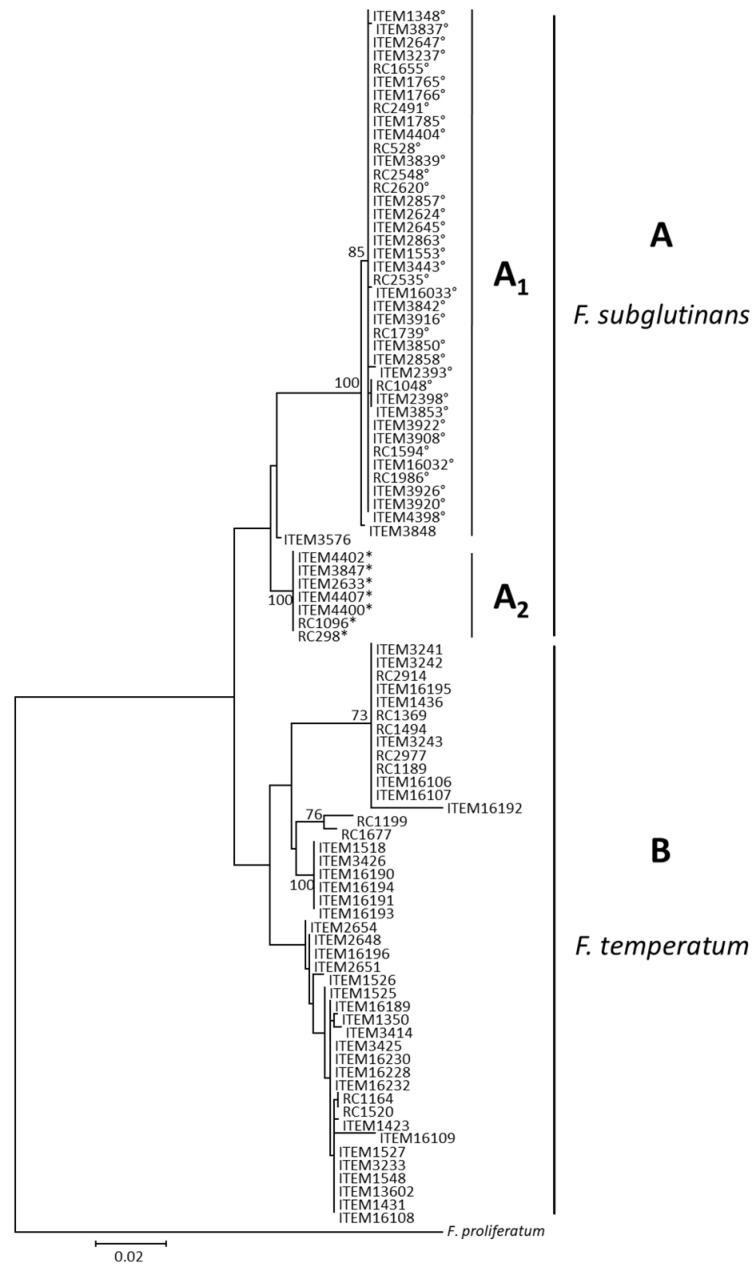
Phylogenetic tree based on the combined sequences (1022 bp) of SNP298 and SNP528 regions. Data were obtained from 93 strains. Sequences were aligned using MUSCLE as implemented in MEGAX. The evolutionary history was inferred using the maximum likelihood method as implemented in IQ-Tree, with the substitution model K2P + R2. Numbers on branches indicates bootstrap values based on 1000 pseudoreplicates. The tree is rooted with *F. proliferatum* (NRRL62905) according to Fumero et al.’s (2020) study [13]. *: strains with SNP298; °: strains with SNP528.

**Figure 5:**
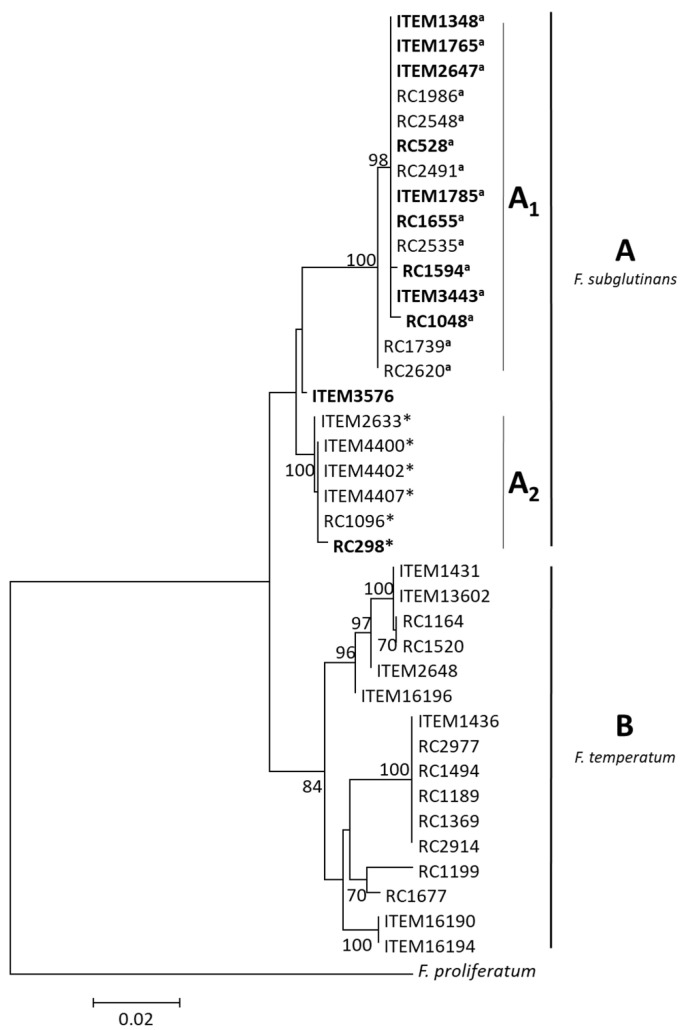
Phylogenetic tree based on the combined sequences (1671 bp) of SNP298, SNP528, and NRPS22ins. Data were obtained from a subset of 39 strains. Sequences were aligned using MUSCLE as implemented in MEGAX. The evolutionary history was inferred using the maximum likelihood method as implemented in IQ-Tree, with substitution model K2P + G4. Numbers on branches indicate bootstrap values based on 1000 pseudoreplicates. The tree is rooted with *F. proliferatum* (NRRL62905) according to Fumero et al.’s (2020) study [13]. ^a^ strains with SNP528; * strains with SNP298; Bold: strains with 184 bp insertion.

We analyzed the variation in the three selected loci of Bea1 within a subset of isolates from our collection by performing phylogenetic analyses. Two datasets were analyzed: (i) concatenated SNP298-SNP528 (1022 bp) for 92 strains (Figure 4) and (ii) concatenated NRPS22ins-SNP298-SNP528 (1671 bp) for 38 strains (22 *F. subglutinans* and 16 *F. temperatum*) (Figure 5) holding 184 bases in the coding region.

The phylogenetic tree of the SNP298-SNP528 combined data set was concordant with the species phylogeny, resulting in two distinct clades: strains belonging to *F. subglutinans* (clade A) and *F. temperatum* (clade B), respectively (Figure 4).

However, unlike Tef1′s phylogeny, those clades did not have significant bootstrap support. Moreover, while in Tef1′s phylogeny most of the *F. subglutinans* isolates were resolved in one subclade (Fs1) and only two isolates were grouped in the subclade Fs2, the SNP298-SNP528 phylogeny was fixed in isolates carrying SNP528 in subclade A1 (bootstrap 100) and the isolates carrying SNP298 in subclade A2 (bootstrap 100). Finally, ITEM 3848 and ITEM 3576 were fixed within subclade A1 and into a clade independent of the other subclades, respectively. The clade B including the *F. temperatum* strains showed more variability than in species phylogeny, although such variability seems not to be correlated with beauvericin biosynthesis.

The phylogenetic tree of the NRPS22ins-SNP298-SNP528 combined data set was concordant with the topology showed in Figure 4 (Figure 5).

*F. temperatum* strains were contained within a well-supported clade (bootstrap value, 84), while clade A, including *F. subglutinans* strains, did not have significant bootstrap support. Within clade A, two well-supported sublades A1 and A2 included isolates with SNP528 and with SNP298, respectively, while ITEM 3576 was fixed in a clade independent of the other subclades.

## 4. Discussion

Ensuring a reliable assignment of mycotoxin profiles to fungal species is crucial for better insight into the toxicological risk associated with crop diseases. Over the last two decades, increasing effort has been made to analyze species concepts and the related mycotoxin biosynthesis using genome and metabolome data. Among the *Fusarium* species complexes, the FFSC has undergone a notable expansion and several reassessments, as species initially identified by morphological tools have been reevaluated with biological species concepts, through in vitro mating tests, and molecular criteria, defining new phylogenetic-based boundaries, like that between *F. subglutinans* and *F. temperatum*.

Unlike the recognition based on morphological and biological species concepts, the phylogenetic approach led to the description of these entities as two distinct species [11]. The *Tef1* phylogeny sorted the set of strains analyzed in this study into two well-supported clades, corresponding to *F. subglutinans* and *F. temperatum*, respectively. Although several studies distinguished these entities using sequences of multiple phylogenetically informative loci [11,12,13], the 680 bp 5′ portion of *Tef1* gene was confirmed to be highly informative at the taxonomic level, supporting the trend of the research community to adopt this gene as the primary barcoding locus in the *Fusarium* genus [8,22]. Furthermore, this nucleotide variability, related to taxonomical boundaries, has been exploited for the development of two species-specific primer pairs, usable indifferently and singularly, when discriminating between *F. subglutinans* and *F. temperatum*, because both of them amplify only target species and not the non-target species. The application of TEF1-based markers in routine diagnostic assays can significantly improve the monitoring of *Fusarium* populations in agricultural crop management, ensuring timely interventions of fungal infections in crops. This integrated molecular approach enhances the ability to track and mitigate the risk of mycotoxin contamination in maize and other susceptible crops.

Genomic and metabolomic data found that both species show a species-specific chemotype based on patterns of mycotoxin [10,13]. *F. subglutinans* can produce fusaproliferin and moniliformin, while *F. temperatum* can produce beauvericin, fusaproliferin and moniliformin. Although the genomes of two *F. subglutinans* isolated from maize in Argentina carry functional *Bea2* to *Bea4* genes, the *Bea1* gene contains the 184 bp insertion and SNPs, likely responsible for the non-functionality of the encoded NRPS. In this study, we have investigated whether those mutations were spread among *F. subglutinans* strains recovered from diverse geographic and climatic origins and whether the 184 bp insertion, predicted as a novel intron in the in silico analysis, was retained in the transcripts from the mutated gene.

The analysis of SNPs (SNP528, NRPS22ins, and SNP298) in the *Bea1* gene provides a practical method for differentiating strains that are capable or not of producing beauvericin. The screening across a large collection of strains belonging to *F. subglutinans*. demonstrated the robustness of these markers for large-scale applications. In contrast, *F. temperatum* strains did not exhibit any mutations in the analyzed regions of the *Bea1* gene that could differentiate BEA producers from non-producers, suggesting that the regulation of beauvericin production in *F. temperatum* may likely involve other genetic loci or non-genetic factors. Our findings revealed that the nucleotide mutations identified in the two genomes of *F. subglutinans* from Argentina, RC298 and RC528 [13], were distributed across the strains of the same species isolated worldwide. SNP528, which introduces a premature stop codon 1907 bp upstream of the 3′ end of the coding region, was the most common mutation, being found in 39 out of 48 *F. subglutinans* strains, followed by NRPS22ins (35 strains) and SNP298 (in 7 strains). Co-occurrence of two mutations was not found in all *F. subglutinans* strains, but the most common occurrence was the combination of NRPS22ins and SNP528 (in 33 strains), while the presence of NRPS22ins and SNP298 was found only in the reference genome (RC 298) from Argentina. This result might suggest that NRPS22ins likely occurred first, as it is widely distributed across *F. subglutinans* strains from different geographical origins. Moreover, it was found in combination with both SNP298 and SNP528, while these latter mutations were never found together, leading us to suppose that they accumulated later in the *Bea1* coding region to inactivate BEA production. However, both SNPs were also found individually, even though their distribution was restricted to limited geographical areas, such as Argentina (SNP528, combination V) and Argentina, Serbia, Slovakia, and the USA (SNP298, combination IV). The observation that *F. subglutinans* strains carrying SNP528 and the SNP298 clustered in two well-distinct clades, regardless of the presence or absence of NRPS22ins, led us to suppose that those non-synonymous SNPs occurred at different times in different regions, and that the resulting inactivation of BEA production has caused the accumulation of other synonymous and non-synonymous SNPs, including NRPS22ins, in the entire gene-coding region. The absence of nucleotide variabilities (combination VI) in only one *F. subglutinans* strain (ITEM 3576) suggests that there could be another process of evolution, and other mutations in *Bea1* or elsewhere can be found, possibly preventing beauvericin biosynthesis. Our phylogenetic analysis based on the combined SNP298-SNP528 data set clearly sorted *F. subglutinans* and *F. temperatum* into two distinct clades. This suggests a stepwise accumulation of mutations in *Bea1* during the divergence of the two species. Understanding the evolutionary history of these mutations not only sheds light on the molecular mechanisms of beauvericin inactivation but also highlights the potential for using genetic markers as indicators of evolutionary and functional traits in fungal populations. The phylogenetic data thus reinforce the utility of these markers in distinguishing *Fusarium* species based on their beauvericin production capabilities.

In this study, we also analyzed the presence/absence of the additional 184 bp by sequencing *Bea1* transcripts from different *F. subglutinans* belonging to combination I, II, and III. The presence of insertion was seen in the transcripts from all the *F. subglutinans* strains included in the analysis, revealing that it is not considered as an intron, as predicted in the in silico analysis, and resulting in a protein projected to be nonfunctional.

In conclusion, all BEA-nonproducing strains have revealed at least one of the three tested DNA variations in *Bea1*, even though not always in the same strain, leading us to hypothesize that after divergence of the two species, multiple mutations have occurred in the *Bea1* gene of *F. subglutinans*, which has been maintained and are responsible for the inability to produce BEA. In BEA-nonproducing *F. temperatum* strains, the cause of inability to produce BEA could be related to some other locus or loci, and again nucleotide mutation should have appeared after differentiating by the *F. subglutinans* species.

Overall, the hypothesis discussed in our previous work [Fumero et al., 2020] [13] was confirmed in this study, according to which the presence of a few non-synonymous SNPs in *Bea1*, as well as the absence of loss-of-function mutations in the other *Bea* genes, suggest that the process of BEA inactivation in *F. subglutinans* began relatively recently.

It is interesting that *Bea1* is located near the end of chromosome 9, where enhanced intraspecific genetic diversity and polymorphisms have been recently observed [Fumero et al., 2021] [14]. Moreover, subtelomeric regions are enriched in putative host–pathogen interaction genes and ecological niche adaptation. It is possible that potential factors involved in the process of speciation and divergence, such as climate and subtle host–plant differences, may have driven the different pathogenicity pattern of this closely related species, making BEA production unneeded in the *F. subglutinans* strains commonly isolated from warmer and drier regions [10,14,23,24].

In conclusion, our study highlights the significant genomic differences between *Fusarium subglutinans* and *F. temperatum*. While *F. subglutinans* harbors several mutations in the *Bea1* gene, leading to the inactivation of beauvericin production, *F. temperatum* did not show any mutations in the analyzed regions of the *Bea1* gene. This suggests that different genetic mechanisms regulate beauvericin production in these two species. Understanding these differences is crucial for developing effective diagnostic tools and improving crop management strategies. The PCR tools developed as part of this study can be used to rapidly differentiate between strains of *F. subglutinans* and *F. temperatum* in maize crops, enabling more targeted and effective crop management strategies. This approach provides a valuable step towards the rapid identification and management of *Fusarium* species in agricultural settings.

## Figures and Tables

**Table 1:** Species-specific primers designed in the *TEF1* gene.

Primers	5′ > 3′ Sequence	Tm (°C)	Exp. Size (bp)	Species Target
subF	GCGTTTCTGCCCTCTCATTTT	59	71	*F. subglutinans*
subR	TCGGCGGCTTCCTATTGTT			
tempF	TTTCTGCCCTCCCATTGC	59	68	*F. temperatum*
tempR	GCTCAGCGGCTTCCTATTGAC			

**Table 2:** Sequence of oligonucleotide primers designed in the *Bea1* gene.

Primers	5′ > 3′ Sequence	Tm (°C)	Expected Size (bp)
Nrps22insF	TTTCCGCGCAGCACACTAT	58	710/526 *
Nrps22insR	AACATTCGGCTTCTCAAGACCA		
SNP298F	GAGGCCCAGAATCTGATTCG	58	554
SNP298R	TGGTTATCATTCGCGTCGC		
SNP528F	CAATCGTCCGCTACAGGSA	58	656
SNP528R	ACCACTATCGTAGCATCYARTCG		

* with/without additional 184 bp region.

**Table 3:** Distribution of nucleotide variabilities in the *Bea1* gene among *F. subglutinans* strains and the relative combinations.

NRPS22 Loci			Combination	Geographic Origin	*F. subglutinans* Strains
NRPS22ins	SNP298	SNP528			
+	−	−	I	USA	ITEM 3848
+	+	−	II	Argentina	RC 298
+	−	+	III	Argentina Germany Italy Poland Serbia Slovakia USA	ITEM (1348-1553-1765-1766-1785-2393-2398-2624-2645-2647-2857-2858-2863-3237-3443-3837-3839-3842-3850-3853-3908-3916-3920-3922-3926-4398-4404-16032-16033) RC (1048-1594-1655-528)
−	+	−	IV	Argentina Serbia Slovakia USA	ITEM (2633-3847-4400-4402-4407) RC 1096
−	−	+	V	Argentina	RC (1739-1986-2491-2535-2548-2620)
−	−	−	VI	Germany	ITEM 3576

+: presence of headed variation. −: absence of headed variation.

## Data Availability

The raw data supporting the conclusions of this article will be made available by the authors on request.

## Supplementary Materials

The following supporting information can be downloaded at: https://www.mdpi.com/article/10.3390/jof10110785/s1, Table S1: Distribution of nucleotide variability in three loci of *bea1* (NRPS22) among 48 *F. subglutinans* and 44 *F. temperatum* strains collected worldwide.
